# Arecoline Induces an Excitatory Response in Ventral Tegmental Area Dopaminergic Neurons in Anesthetized Rats

**DOI:** 10.3389/fphar.2022.872212

**Published:** 2022-04-25

**Authors:** Qinghui Lan, Peiqing Guan, Chunzheng Huang, Shile Huang, Peiling Zhou, Changzheng Zhang

**Affiliations:** ^1^ School of Educational Sciences, Lingnan Normal University, Zhanjiang, China; ^2^ Western Guangdong Characteristic Biology and Medicine Engineering and Research Center, School of Chemistry and Chemical Engineering, Lingnan Normal University, Zhanjiang, China

**Keywords:** arecoline, ventral tegmental area, dopaminergic neuron, GABAergic neuron, electrophysiological activity

## Abstract

Arecoline is the principle psychoactive alkaloid in areca nuts. Areca nuts are chewable seeds of *Areca catechu* L., which are epidemic plants that grow in tropical and subtropical countries and cause dependency after long-term use. However, the mechanisms underlying such dependency remain largely unclear, and therefore, no effective interventions for its cessation have been developed. The present study aimed to examine the effects of arecoline on neurons of the ventral tegmental area (VTA). After rats were anesthetized and craniotomized, electrophysiological electrodes were lowered into the VTA to obtain extracellular recordings. The mean firing rate of dopaminergic and GABAergic neurons were then calculated and analyzed before and after arecoline treatment. The burst characteristics of the dopaminergic neurons were also analyzed. The results showed that arecoline evoked a significant enhancement of the firing rate of dopaminergic neurons, but not GABAergic neurons. Moreover, arecoline evoked remarkable burst firings in the dopaminergic neurons, including an increase in the burst rate, elongation in the burst duration, and an enhancement in the number of spikes per burst. Collectively, the findings revealed that arecoline significantly excited VTA dopaminergic neurons, which may be a mechanism underlying areca nut dependency and a potential target for areca nut cessation therapy.

## Introduction

Arecoline is the main psychoactive alkaloid in areca nut, which is the chewable seed of *Areca catechu* L. *Areca catechu* L. is a widely popular plant and is a traditional herbal medicine in China, India, Southeast Asia, the East African seaboard, and the Western Pacific (Peng W et al., 2015; Volgin et al., 2019; Duguet et al., 2020). It has been estimated that there are over 600 million global users of areca nut products, making areca nut the fourth most popular psychoactive substance after tobacco, alcohol, and caffeine (Giri et al., 2006).

Arecoline is a natural cholinomimetic drug that evokes a wide spectrum of pharmacological effects on the nervous, cardiovascular, digestive, and endocrine systems, and also has anti-parasitic effects (Peng W et al., 2015; Peng W et al., 2015; Liu et al., 2016). Arecoline exhibits multiple adverse effects, including growth retardation, developmental defects, locomotive impairment, cardiovascular disorders, and hyporeflexia, as shown in studies of zebrafishes, mice, rats, and humans (Pichini et al., 2005; Peng W. H et al., 2015; Peng et al., 2016; Dasgupta et al., 2017; Dasgupta et al., 2018; Winger, 2021). Arecoline induces severe toxicity on cellular and molecular levels, such as oral submucosal fibrosis, oral squamous cell carcinoma, and genotoxicity (Liu et al., 2016; Drake and Pjh, 2018).

Nevertheless, chewing areca nuts causes relaxation, alertness, elation, and mild euphoria, which leads to user dependencies. Discontinuation in dependent individuals produces withdrawal syndromes, including lethargy, anxiety, irritability, and insomnia (Abbas et al., 2013; Serikuly et al., 2020). The dependency rate is 20–90% among areca nut users (Ko et al., 2020); however, the neurobiological mechanism underlying dependency is not fully understood, and thus, no effective cessation therapies have been developed.

Considering that ventral tegmental area (VTA) dopaminergic neurons play important roles in addiction, in this study, we performed an electrophysiological experiment to determine whether and how arecoline modulates VTA neuron activities. The findings suggested that arecoline induced dopaminergic hyperactivity, including increases in the firing rate and induction of the burst firing model (a periodic high-discharge rate pattern), which may contribute to areca nut addiction.

## Materials and Methods

### Animals

Fifty-four male adult Sprague-Dawley rats (2–3 months of age, weighing 240 ± 20 g) were obtained from Guangdong Medical Laboratory Animal Center (Foshan, China). Animals were housed in a temperature- (23 ± 1°C) and light-controlled (12/12-h light/dark cycle) environment, with rodent food and clean water *ad libitum*. All animal experiments abided by the rules of the National Institutes of Health Guide for the Care and Use of Laboratory Animals (NIH Publication No. 80–23, revised in 1996) and were approved by our University’s Academic and Ethics Committee. All efforts were made to minimize the number of animals used, as well as their suffering.

### Surgical Procedure

The rat brain surgical procedure followed that described in multiple reports. Briefly, rats were anesthetized with isoflurane (RWD Life Science Co. Ltd., Shenzhen, China) and mounted onto a stereotaxic instrument (RWD Life Science Co., Ltd.) for craniotomy on one side of the VTA (x: -4.92–5.20; y: ±0.6–1.0; z: 8.3–8.8), according to a rat brain atlas (Paxinos and Watson, 2007). The single tungsten electrodes ( ∼ 30 μm in diameter with ∼ 500 kΩ in impedance; Alpha Omega Engineering, Nof HaGalil, Israel) were slowly lowered into the VTA using a computer-controlled stepper motor (IVM-1000; Scientifica, East Sussex, UK) for extracellular recordings.

The electrophysiological signals were acquired and recorded by a signal collecting and processing apparatus (AlphaLab SnR, Alpha Omega Engineering). The analog signals were amplified and band-pass filtered between 0.3 and 9 kHz, at a spike sampling rate of 44 kHz. Once the neuronal firings were stable for at least 20 min, the rats were injected with saline (0.9% NaCl/0.2 ml; *n* = 24) or arecoline (0.2 mg/kg/0.2 ml; *n* = 30) through the caudal vein. Approximately 30 min after the saline/arecoline treatment, rats were intraperitoneally injected with the D2 receptor agonist, quinpirole (2 mg/kg/1.0 ml; Santa Cruz Biotechnology, CA, USA) to distinguish putative dopaminergic and GABAergic neurons (Burkhardt and Adermark, 2014).

At the conclusion of the experiment, DC current was passed through the electrodes (300 μA, 5 min), and rat brains were processed into coronal sections using a freezing microtome (CM 1860, Leica Instruments, Mannheim, Germany) for histological evaluations of the recoding sites (Paxinos and Watson, 2007).

### Neuronal Firing Measurements

The action potentials were sorted using Spike2 software (Version 8.0, CED Ltd., Cambridge, UK) and analyzed using NeuroExplorer software (Version 5, Plexon Inc., Dallas, USA). Putative dopaminergic and GABAergic neurons were distinguished using the following criteria (Jin and Costa, 2010; Li et al., 2012; Tolu et al., 2012; Burkhardt and Adermark, 2014): dopaminergic neurons had low spontaneous firing rates (<10 Hz) and inhibitory responses (>50%) to quinpirole (a D2 receptor agonist), whereas the putative GABAergic neurons had high spontaneous firing rates (>10 Hz) and irresponsiveness to quinpirole stimulus. The mean firing rates of the dopaminergic neurons were calculated over 60 s time windows before (beginning at 120 s prior to arecoline injection) and after (during the peak response sequence) arecoline treatment. As there were no firing alterations in the dopaminergic neurons following saline treatment, or to GABAergic neurons following arecoline and saline stimuli, the mean firing rates for those groups were calculated from a 60-s firing sequence before (beginning at 120 s prior to injection) and after (beginning at 200 s after injection) drug treatment. The neuronal firing responses to quinpirole were measured from a 60-s firing sequence about 2 min before and after quinpirole treatment.

The burst analysis of dopaminergic neurons was performed over the same 60 s time widow before and after arecoline/saline administration. A spike burst was defined as a minimum of three successive spikes with an initial interspike interval (ISI) ≤ 80 ms and a termination ISI >160 ms (Grace and Bunney, 1984; Chen and Lodge, 2013). The burst rate, the burst duration, and the number of spikes per burst of the putative dopaminergic neurons before and after arecoline/saline treatments were calculated and analyzed.

### Statistical Analysis

All data are presented as the mean ± standard error of the mean. A two-way analysis of variance (ANOVA) followed by a Fisher’s least significant difference *post hoc* test was conducted for statistical analyses, and the *t*-test was used as needed. A *p* < 0.05 was considered statistically significant for all tests.

## Results

### Arecoline Excites Dopaminergic Neurons, but Not GABAergic Neurons

**FIGURE 1:**
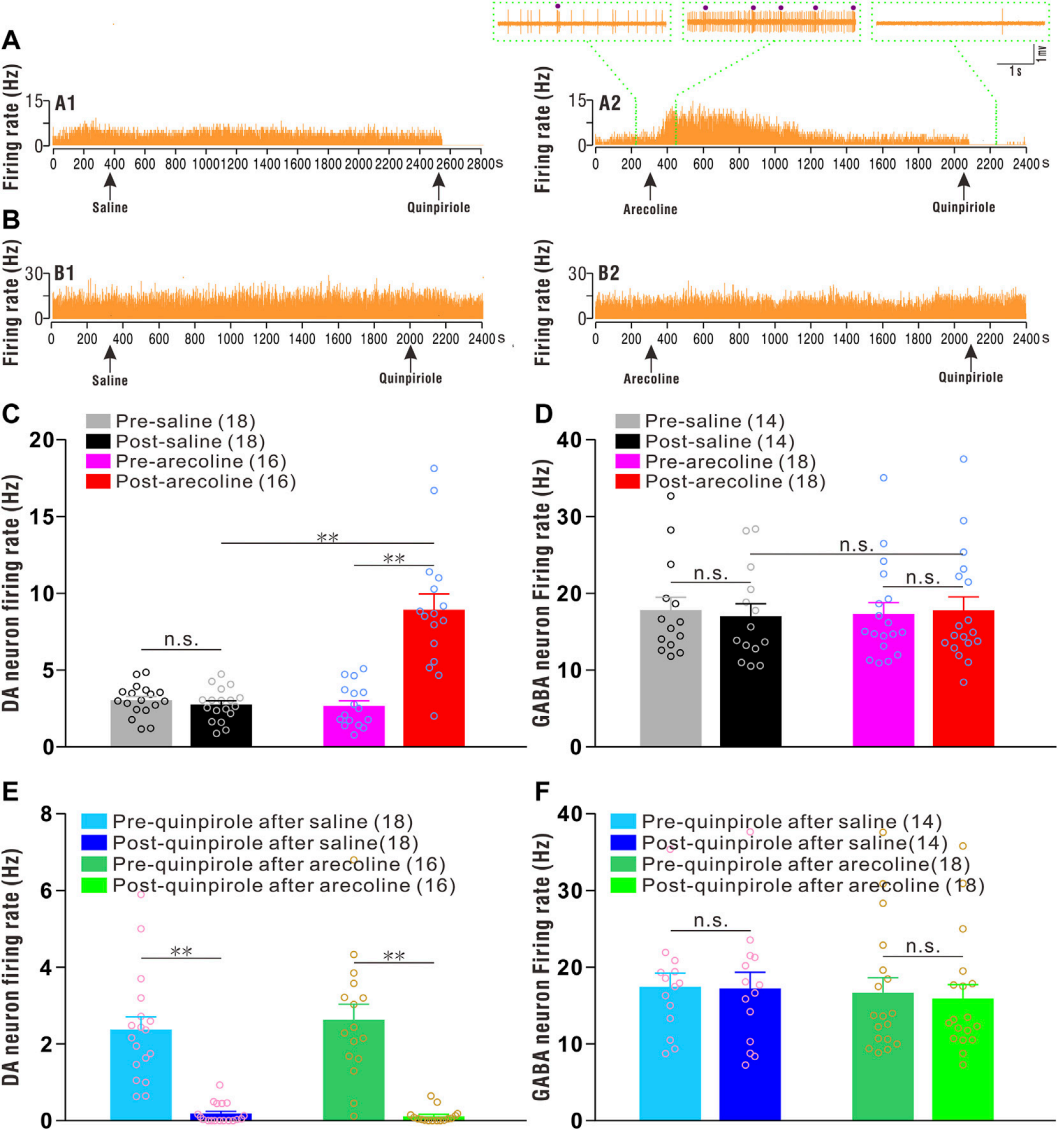
Arecoline increases the firing rates of VTA dopaminergic neurons. **(A)** Representative recordings from a dopaminergic neuron before and after saline (A1) and arecoline (A2) treatment, and then with quinpirole administration. **(B)** Representative recordings from a GABAergic neuron before and after saline (B1) and arecoline (B2) treatment, and then with quinpirole administration. The arrowheads indicate the time of injections. The left and middle insets (above A2) represent the traces of dopaminergic neuron firings before and after arecoline administration, and the right inset (above A2) represents the firing response to quinpirole. The small dots indicate putative bursts. **(C,D)** Histograms of the firing rates of dopaminergic neurons **(C)** and GABAergic neurons **(D)** before and after saline/arecoline injections. **(E,F)** Histograms of the firing rates of dopaminergic neurons **(E)** and GABAergic neurons **(F)** before and after quinpirole injections. The numbers presented in the parentheses denote the number of neurons measured, while the overlaid dots are the individual data points. DA neuron: dopaminergic neuron. ^**^ *p* < 0.01; NS, not significant.

Thirty-four putative dopaminergic neurons were identified and sorted from the saline (*n* = 18 cells/15 rats) and arecoline treated groups (*n* = 16 cells/15 rats). Before arecoline/saline injection, the basal firing rates of the putative dopaminergic neurons ranged from 0.77 to 5.08 Hz, with an average of 2.86 ± 0.20 Hz, which showed significant inhibitory responses to quinpirole stimuli (Figures 1A,C,E). A two-way ANOVA revealed the significant effects of drug (arecoline and saline; F1,32 = 26.781, *p* < 0.001), time (before and after treatments; F1,32 = 32.346, *p* < 0.001), and drug × time interactions (F1,32 = 38.872, *p* < 0.001) among the groups. The firing rate was significantly increased after arecoline treatment (by 2.36-fold *vs* before injection; *p* < 0.01; Figure 1C), whereas the firing rates exhibited no obvious alterations following vehicle treatment (*p* = 0.411; Figure 1C).

Thirty-two putative GABAergic neurons were identified from saline (*n* = 14 cells/12 rats) and arecoline treatments (*n* = 18 cells/15 rats). Before arecoline/saline treatment, the firing rate of the putative GABAergic neurons was 17.53 ± 1.10 Hz (ranging from 10.93 to 35.05 Hz), which indicated that they were insensitive to quinpirole administration (Figures 1B,D,F). A two-way ANOVA revealed no significant effect of drug (F1,30 = 0.003, *p* = 0.954), time (F1,30 = 0.141, *p* = 0.710), and drug × time interactions (F1,30 = 2.224, *p* = 0.146) among the groups, thus, indicating that neither arecoline nor saline affected the firing activities of the GABAergic neurons.

The above results indicated that arecoline induced a significant excitatory response in VTA dopaminergic neurons, but exerted no effect on the GABAergic neurons.

### Arecoline Induces Remarkable Burst Firings in Dopaminergic Neurons

**FIGURE 2:**
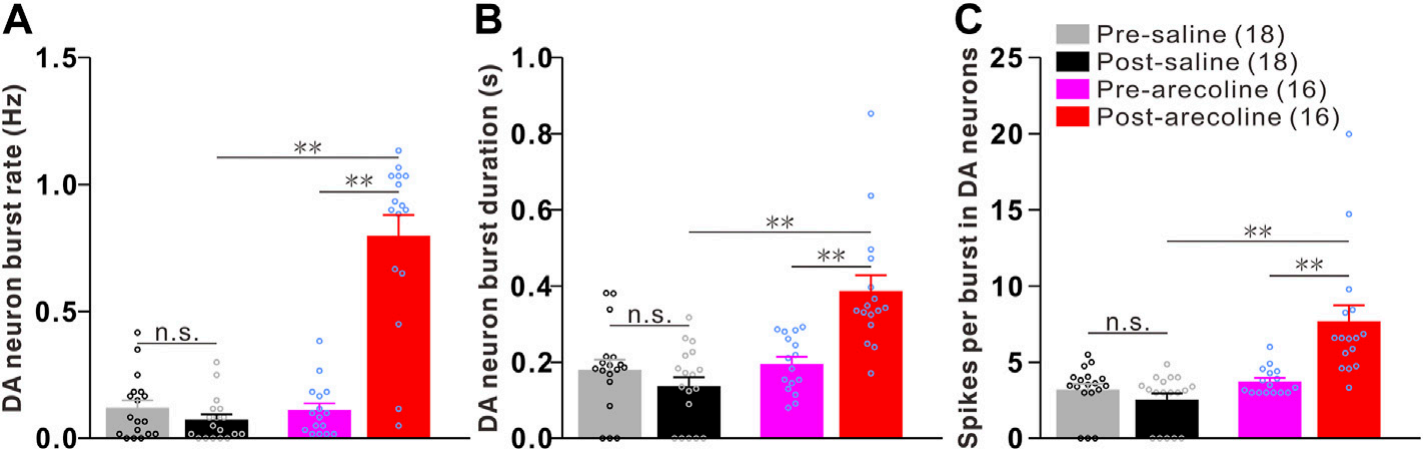
Arecoline induces burst firings in VTA dopaminergic neurons. **(A–C)** Histograms of the burst parameters in dopaminergic neurons before and after saline/arecoline treatments. **(A)** Burst rate, **(B)** the burst duration, and **(C)** the number of spikes per bursts. The numbers presented in the parentheses denote the number of neurons measured, while the overlaid dots are the individual data points. DA neuron: dopaminergic neuron. ^**^ *p* < 0.01; NS, not significant.

Arecoline treatment significantly increased the burst rate in dopaminergic neurons. Two-way ANOVA revealed a significant effect of drug (F1,32 = 56.483, *p* < 0.001), time (F1,32 = 57.713, *p* < 0.001), and drug × time interactions (F1,32 = 76.027, *p* < 0.001) among the groups. The burst rate was significantly increased by 6.27-fold after arecoline treatment in comparison to the burst rate before treatment (*p* < 0.01; Figure 2A), whereas no significant changes were observed following vehicle treatment (*p* = 0.196; Figure 2A).

Similarly, the burst duration in dopaminergic neurons showed effects on drug (F1,32 = 20.349, *p* < 0.001), time (F1,32 = 7.167, *p* < 0.001) and drug × time interactions (F1,32 = 18.014, *p* < 0.001), in which burst duration was significantly increased following arecoline (*p* < 0.01 *vs* before injection) but not saline treatment (*p* = 0.236 *vs* before injection; Figure 2B). The number of spikes per burst in dopaminergic neurons also showed effects on drug (F1,32 = 21.914, *p* < 0.001), time (F1,32 = 8.580, *p* = 0.006) and drug × time interactions (F1,32 = 16.847, *p* < 0.001), in which the spike number per burst was significantly increased due to arecoline stimulus (*p* < 0.01 *vs* before injection), whereas no obvious changes appeared after vehicle treatment (*p* = 0.240 *vs* before injection; Figure 2C). Those findings demonstrated that arecoline evoked remarkable enhancements in the burst activities in VTA dopaminergic neurons, including an increase in the burst rate, an elongation of the burst duration, and an increase in the spikes per burst.

**FIGURE 3:**
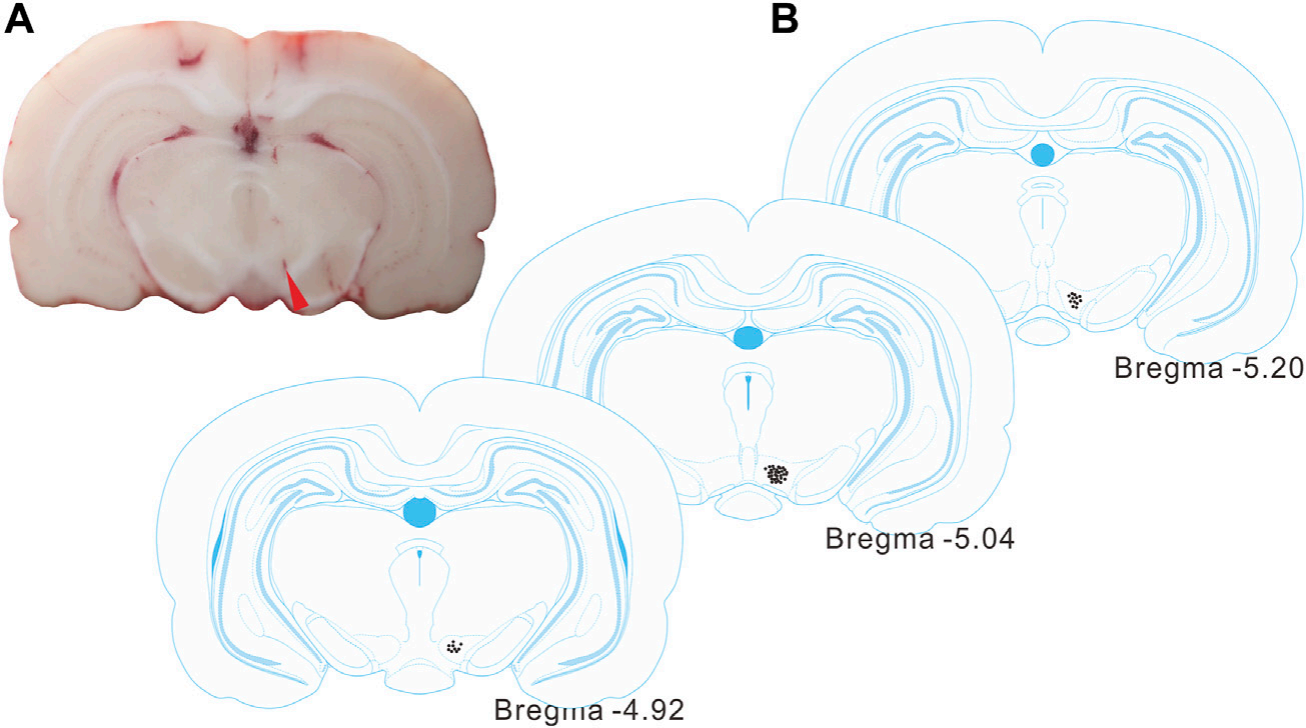
Identification of the recording site in the VTA. **(A)** A representative histological section from a rat brain showing the electrode site (red arrowhead). **(B)** Histological reconstructions showing the recording sites in VTAs (between -4.92 and -5.20 mm from bregma) according to the rat brain atlas.

Following the recordings, the electrode sites were identified by histological observations (Figures 3A,B), and were contained within the VTA regions, according to the rat brain atlas (Paxinos and Watson, 2007).

## Discussion

Growing evidence suggests that individuals who chew areca nuts exhibit altered brain functional connectivity (for example, higher functional connectivity between the temporal, parietal, and frontal areas) (Sariah et al., 2020). The results from functional magnetic resonance imaging revealed the neurological mechanisms of the brain reward systems (Ko et al., 2020). The mesocorticolimbic system (*e.g.,* VTA, nucleus accumbens, and prefrontal cortex) plays a primary role in processing rewards, and dopaminergic neurons in the VTA are considered to play a pivotal role in drug dependence (Morgane et al., 2005; Volkow and Morales, 2015). It is believed that VTA hyperdopaminergia is an important contributor to substance addiction. Several lines of evidence have demonstrated that arecoline administration or areca nut chewing elevated brain dopamine levels (Molinengo et al., 1986); however, to our knowledge, the electrophysiological properties of dopaminergic neurons following arecoline exposure have not been examined.

The VTA is primarily comprised of dopaminergic neurons (60–65% of the total number of the neurons) and GABAergic neurons (30–35% of the total number of neurons) (Morales and Margolis, 2017; Zhang et al., 2018). In the present study, we found that the intravenous injection of arecoline induced a significant increase in the firing activities of VTA dopaminergic neurons, but not GABAergic neurons. It is reported that VTA has a small portion (accounting for 2–3% in the total neurons) of glutamatergic neurons (Yamaguchi et al., 2007; Morales and Margolis, 2017), which also deserves further investigation whether they response to arecoline stimulus.

Dopaminergic neurons exhibit tonic and burst firing models. Tonic firing produces physiological dopamine release that sends “safety” signals downstream, whereas burst firing causes abundant dopamine release that is associated with reward-seeking behaviors (Zhang et al., 2018; Zhou et al., 2021). In our present study, we found that arecoline significantly enhanced the firing rates and burst activities of VTA dopaminergic neurons, but exerted no significant effects on GABA neuronal firings, which was similar to the effects of cocaine on the firing characteristics of the dopaminergic and GABAergic neurons (Creed et al., 2016). Increased dopaminergic neuron activities will enhance extrasynaptic dopamine levels, and the burst firings in particular markedly promote synaptic dopamine release (Floresco et al., 2003), which is strongly associated with substance dependence (Zhang et al., 2018; Jing et al., 2022). Therefore, arecoline-induced VTA dopamine neuron excitation may, to some extent, associate with the addictive phenotypes of areca nut users.

The arecoline dosage used in the present study was 0.2 mg/kg (administered *via* intravenous injection), which was far below the lethal dose (Sahoo et al., 2018). However, our reports, as well as those of others, have demonstrated that a low dose of arecoline was sufficient to induce cardiovascular effects (Deng et al., 2020) or behavioral manifestations (Winger, 2021). Low doses of arecoline induced a significant increase in neuronal activity, suggesting that dopaminergic neurons were sensitive to arecoline stimulus.

Despite the findings in this preliminary study, some shortcomings must be addressed in future investigations. First of all, only male rats were used in the study to avoid the confounding influences of sex, especially hormonal changes on dopamine activity, for example female rats showed different dopamine uptake and with fluctuation characteristics during female estrous cycle relative to males (Morissette and Di Paolo, 1993), whereas an in-depth exploration of sex differences in arecoline-evoked dopamine neuronal activities would provide additional insights into addiction among areca nut users. Second, as arecoline has muscarinic and nicotinic receptor agonist properties, and both receptors exhibit modulatory effects on dopaminergic neurons (Papke et al., 2015; Horenstein et al., 2019), it is necessary to explore which receptors (or which subtypes) mediate arecoline-induced excitation in dopaminergic neurons. Additionally, considering that arecoline has several metabolites (Giri et al., 2006), it is necessary to determine whether such metabolites are also involved in the dopaminergic excitatory response.

In conclusion, the current study demonstrated that arecoline, the major alkaloid of areca nut, induced an excitatory response in VTA dopaminergic neurons, increasing the firing and burst rates. The findings provided a novel mechanism leading to areca nut addiction, and suggested a potential therapeutic target for the clinical intervention of areca nut dependence.

## Data Availability

The raw data supporting the conclusions of this article will be made available by the authors, without undue reservation.
